# New Appliances and Things Medical

**Published:** 1903-05-16

**Authors:** 


### NEW APPLIANCES AND THINGS MEDICAL.

[We shall be glad to receive, at our Office, 28 & 29 Southampton Street, Strand, London, W.C., from the manufacturers, specimens of all new preparations and appliances which may be brought out from time to time.]

### DR. KISSLING'S NON-NICOTINIC CIGARS.

(Dr. Kissling's Agency, 15 Fore Street, London, E.C.)

The flavour of a cigar no more depends on the nicotine which it contains than does the bouquet of a vintage claret on its alcoholic contents. As is well known with regard to wines, the flavour or aroma depends on the volatile ethers, and so with regard to tobaccos, it is not the character of the nicotine which makes one sample excellent and another bad, but certain aromatic bodies which are chemically difficult to separate and define. Nicotine, however, has narcotic and poisonous properties, and it is to these properties that the pernicious effects of excessive smoking are chiefly due. Nicotine has a selective affinity for nerve structures: for instance, tobacco amblyopia, colour blindness, and amaurosis are not uncommon results of poisoning of the optic nerves by nicotine poisoning; and irregularity and irritability of the heart's action are frequent consequences of over-smoking. The advantages, therefore, of being able to enjoy a fragrant cigar without fear of any of the ill effects of nicotine poisoning should be, to say the least, a great boon. Dr. Kissling has invented a process of freeing tobacco of the greater proportion of the contained nicotine without materially altering the aroma or flavour of the tobacco itself, and his non-nicotine cigars are the result of his invention. We strongly advise sufferers from nicotine poisoning, whether they be dyspeptics, the semi-blind, or the owners of irritable hearts, to substitute nicotine-free tobacco for the more poisonous varieties to which they owe their troubles.

